# Mitigating urban heat island and enhancing indoor thermal comfort using terrace garden

**DOI:** 10.1038/s41598-024-60546-0

**Published:** 2024-04-27

**Authors:** Girish Visvanathan, Kailas Patil, Yogesh Suryawanshi, Vishal Meshram, Shrikant Jadhav

**Affiliations:** 1 Vishwakarma University, Pune, 411048 India; 2 Vishwakarma Institute of Information Technology, Pune, 411048 India; 3 San Jose State University, San Jose, CA USA

**Keywords:** Engineering, Environmental social sciences

## Abstract

The United Nations advocates for sustainable urban planning and design, emphasizing green infrastructure initiatives to mitigate urban heat island effects and enhance the resilience and livability of cities globally. To address urban heat challenges, a study was conducted in Chennai, India, from April to June 2023. The study focused on assessing temperature dynamics on a building's terrace by comparing a well-maintained garden area with an exposed region. Temperature and humidity sensors were deployed in both the garden and exposed areas of the terrace, as well as within rooms beneath it, to monitor hourly temperature fluctuations. The findings indicate a significant reduction in internal room temperatures in areas with rooftop gardens, ranging from 4 to 11 °C, depending on the time of year and sun's position, compared to rooms with fully exposed roof configurations. Additionally, simulation studies were performed to validate these findings, suggesting that optimizing the distribution of soil beds and plant density across the roof could yield an additional temperature reduction of 3–4 °C, resulting in an overall difference of up to 14–15 °C. The study highlights the efficacy of rooftop gardens in providing cooling effects during daylight hours and maintaining temperature parity post-sunset. Through analysis of sensor data, the research elucidates the intricate relationship between green infrastructure and thermal comfort, offering insights for energy-efficient building design and resilient urban planning. The findings underscore the potential of rooftop gardens in fostering a more comfortable, energy-efficient, and sustainable urban living environment.

## Introduction

Urbanization's global expansion has heightened concerns about the urban heat island (UHI) effect, a phenomenon characterized by increased temperatures in urban areas due to concentrated buildings, extensive paved surfaces, and diminished green spaces. This effect adversely impacts urban life, causing discomfort and compromised thermal comfort, particularly during hot summer months. Beyond human well-being, it escalates energy consumption for cooling, intensifying electricity demand and greenhouse gas emissions.

Addressing the UHI effect has become pivotal in research and urban planning. Terrace gardens, a burgeoning solution, garner attention for their potential to reduce ambient air temperatures, enhance air quality, and augment urban aesthetics. These green spaces also hold promise in improving indoor thermal comfort, offering a natural cooling effect to building interiors.

Effectively implementing and assessing the impact of terrace gardens as a UHI mitigation strategy necessitates comprehensive data on their performance. This involves understanding temperature disparities between garden and exposed areas, especially in diverse weather conditions. Investigating the influence of terrace gardens on indoor thermal comfort is crucial for optimizing energy-efficient building design and fostering sustainable urban environments.

In this study, we present a sensor-based dataset collected from a building terrace with partial garden coverage, capturing temperature variations over a specific period. Additionally, insights from four case studies shed light on the impact of terrace gardens on indoor thermal comfort. The primary rationale for selecting Anbagam shelter is rooted in its status as one of the early adopters of roof gardening in Chennai. This unique characteristic not only aligns with the primary focus on therapeutic and monetary benefits associated with mentally challenged individuals cultivating their own vegetables but also allows for the estimation of the environmental impact of such initiatives.

Anbagam shelter's location in one of the most densely populated areas in Chennai, (with approximately 30,000 people per sq.km. According to the criteria set by the census of India for defining an urban area, this locality meets the minimum population requirement of 5000, has at least 75 percent of the working population engaged in non-agricultural occupations, and exhibits a density of over 400 persons per square kilometre.

The elevated population density reflects a substantial development footprint, with both vertical and horizontal expansion contributing to significant portions of the built environment, including facades and roofs, being exposed to direct sunlight. This high level of urbanization leads to elevated temperatures, commonly associated with the urban heat island (UHI) effect.

Therefore, Anbagam shelter serves as a representative case study for urban concrete jungles experiencing higher-than-normal temperatures due to the UHI effect. The findings from this study have the potential to contribute meaningful insights into mitigating the negative effects of the UHI and promoting thermal comfort. When extrapolated to a larger scale, the outcomes demonstrate the capacity to lower neighbourhood temperatures and enhance urban living conditions in densely populated areas facing similar challenges.

The outcomes of this research offer meaningful insights for urban planners, architects, and building designers considering terrace gardens for UHI mitigation. Moreover, the results aid in estimating energy savings and reducing cooling loads in buildings, contributing to overall energy efficiency. This study contributes to the advancement of knowledge in sustainable urban development, emphasizing the creation of comfortable and resilient urban environments.

The key contributions of this work are outlined below: Comprehensive Sensor-Based Dataset: This study provides a valuable dataset capturing temperature variations between garden areas and exposed regions on a building terrace, offering detailed insights into temperature differentials.

1. *Assessment of terrace gardens on indoor thermal comfort*: The research examines the impact of terrace gardens on indoor thermal comfort, providing insights into their effectiveness in enhancing comfort levels within buildings.
2. *Four case studies*: The study includes four case studies analyzing terrace garden performance under different weather conditions, offering practical insights for real-world applications.
3. *Implications for multiple disciplines*: The findings benefit urban planners, architects, building designers, engineers, botanists, and city/urban planners, informing urban design, energy-efficient building strategies, foliage impact assessment, land surface temperature estimation, and sustainable urban development.
4. *Contribution to sustainable urban development*: The study advances knowledge in urban heat island mitigation, promoting resilient and comfortable urban environments prioritizing energy efficiency and resident well-being.

## Related work

The phenomenon of the urban heat island (UHI) has garnered significant attention in recent years due to its detrimental impact on urban environments. To address this issue and promote urban sustainability, researchers have been investigating various mitigation strategies, one of which involves the implementation of green roofs. In this section, we will review pertinent studies that explore the effectiveness of green roofs in mitigating the UHI effect and fostering environmental sustainability.

Numerous studies have demonstrated the positive influence of green roofs in reducing surface temperatures and alleviating the UHI effect. Akbari and Kolokotsa^1^ conducted an extensive review encompassing three decades of research on UHI and mitigation technologies, highlighting the efficacy of green roofs in tempering urban temperatures. Research gaps in the paper includes the need for updated strategies and technologies in urban heat mitigation and a more comprehensive exploration of the combined effects of tree planting and reflective surfaces. Saadatian et al.^2^ examined the energy aspects of green roofs and their contribution to UHI mitigation, emphasizing their potential in decreasing the demand for cooling energy. The authors suggested the further research include a deeper investigation into the extended energy efficiency of green roofs over time and a more comprehensive examination of their wider environmental consequences.

Getter et al.^3^ explored the carbon sequestration potential of extensive green roofs, emphasizing their role in climate change mitigation and highlighting the need for prolonged assessments of carbon sequestration. Susca^4^ delved into the structural factors of green roofs and their impact on the urban climate, providing valuable insights into UHI mitigation and revealing research gaps that involve more detailed exploration of specific structural elements' influence on cooling performance and the urban microclimate. Getter and Rowe^5^ further discussed the broader role of extensive green roofs in sustainable development, emphasizing contributions to urban biodiversity and storm water management, thereby indicating a need for a deeper investigation into the long-term ecological impacts of extensive green roofs in urban environments. Imran et al.^6^ assessed the effectiveness of green and cool roofs in mitigating the UHI effect during a heat wave, emphasizing cooling benefits and potential energy savings, pointing to research gaps that include further exploration of the long-term performance and energy efficiency of green and cool roofs during extreme heat events. Oberndorfer et al.^7^ examined green roofs as urban ecosystems, delving into ecological structures, functions, and services, revealing gaps in understanding the ecological dynamics and biodiversity maintenance on green roofs that necessitate a more comprehensive exploration. Yang et al.^8^ evaluated UHI mitigation potential in a tropical climate, emphasizing the effectiveness of green and cool roofs in reducing surface temperatures, and highlighting the need for more studies on tropical climate-specific factors influencing the performance of green and cool roofs. Il and Hawkins^9^ employed hardware scale modeling to assess green roofs' impact on UHI mitigation, indicating research gaps that involve a more in-depth exploration of modeling approaches and their accuracy in predicting UHI mitigation outcomes.

Some researchers performed a cost-effectiveness analysis of green roofs, highlighting their long-term benefits and suggesting further economic analyses considering diverse geographical and climatic conditions^10^. The potential of urban forestry, living roofs, and light surfaces in mitigating the UHI effect in New York City, underscoring the need for more studies on the combined impact of multiple mitigation strategies in complex urban environments^11^. The impact of green areas in mitigating the UHI effect, emphasizing their role in adapting to urban heat islands, and indicating research gaps that involve a more detailed examination of specific green area characteristics influencing UHI mitigation^12^. Few authors conducted a surface temperature analysis of an extensive green roof, demonstrating its effectiveness in mitigating the UHI effect in a southern Mediterranean climate, and pointing to research gaps that include a more extensive analysis of the adaptability of green roofs to various Mediterranean subclimates^13^. Another one conducted a review of reflective and green roof mitigation technologies, indicating a need for updated assessments of technological advancements in reflective and green roof technologies^14^. The green roofs are strategies for adapting to urban heat islands, highlighting the potential in reducing surface temperatures and suggesting further exploration of modeling accuracy and the integration of green walls with green roofs for comprehensive UHI mitigation^15^. The Used simulation-based evaluation to assess the impact of green roofs in office building districts, indicating research gaps that involve more studies on the scalability of green roof impact in different urban settings and building types^16^. The artificial neural networks to simulate green roofs' potential in mitigating the UHI effect in Austin, Texas, emphasizing the need for further validation of simulation models and their applicability to diverse urban environments^17^. The impact of green roofs on microclimate and UHI reduction, pointing to research gaps that involve a deeper understanding of microclimate dynamics and specific factors influencing UHI reduction^18^. A socio-spatial analysis of equity in green roofs as a UHI mitigation strategy in Detroit, emphasizing the importance of more comprehensive studies on the social equity implications of green roof implementation in various urban contexts^19^. Conducted a surface heat budget analysis to evaluate the UHI mitigation potential of green roofs, indicating a need for further investigations into the surface heat budget dynamics under different climatic and urban conditions^20^. Passive cooling techniques with green roofs in residential buildings within the Mediterranean region, emphasizing the energy and comfort benefits of green roofs and suggesting more detailed analyses of the specific advantages and limitations of green roofs compared to other passive cooling techniques^21^. The potential of green roofs to reduce building energy consumption and mitigate the UHI effect, indicating a need for a more comprehensive understanding of the interactions between green roofs, building energy consumption, and UHI mitigation outcomes^22,23^.

Furthermore, recent investigations have played a crucial role in advancing our comprehension of the urban heat island (UHI) mitigation capabilities inherent in green roofs. In their study, Su et al.^24^ analyzed the impact of green roofs on outdoor thermal comfort and energy consumption, offering valuable insights into their capacity to improve urban microclimates. Meanwhile, Yu et al.^25^ conducted a case study evaluating the energy and environmental performance of green roofs across diverse climates, emphasizing their positive influence on building energy efficiency and storm water management. Additionally, Ge et al.^26^ explored the cooling effects of green roofs in a subtropical climate, assessing their potential to reduce building energy consumption and enhance outdoor thermal comfort.

Collectively, these reviewed studies underscore the effectiveness of green roofs as a viable strategy for mitigating the UHI effect. They demonstrate that green roofs can contribute to the reduction of surface temperatures, CO2 emissions, and energy consumption, while also improving human thermal comfort and promoting urban sustainability. However, it is essential to consider factors such as climatic conditions, social equity, optimal design, and appropriate plant selection when implementing green roofs for UHI mitigation. While prior research extensively discusses the advantages of urban heat island effect (UHIE) mitigation solutions, a notable gap exists in addressing partial implementation, specifically focusing on a section of a larger roof. Ongoing future initiatives involve sensor-based data collection for air-conditioned spaces, aiming to estimate energy savings through a combination of sensors and Internet of Things (IoT) meters. Additionally, the research explores the use of high-reflectivity fabrics (waterproof) to ensure year-round protection, particularly for vulnerable populations living in steel sheds and hutments. Therefore we planned the study spanning April to June 2023 to analyze urban heat temperature variations between a well-maintained garden area and an exposed region in a building.

## Methods

### Dataset creation

This dataset was generated to estimate the reduction in surface and internal temperatures of areas with rooftop garden or horticulture. The facility where the test was performed consists of differently abled and mentally challenged inmates that spend their time indoors in naturally ventilated spaces. Setting up a rooftop garden was hence a measure to ensure temperature relief to the inmates whilst engaging them in gardening and as a byproduct, enjoy the yields from these gardens. There is a potential to transform 1000s of acres of empty and concrete terrace space in the region to green edible and low albedo infrastructure that can contribute towards heat reduction, improved thermal comfort, and lower energy consumption. Hence, monitoring the implication of one of the terrace gardens set up in a homeless shelter in Otteri, to better understand how this green intervention can be best leveraged for heat reduction at the building level.

### Dataset location

**Figure 1:**
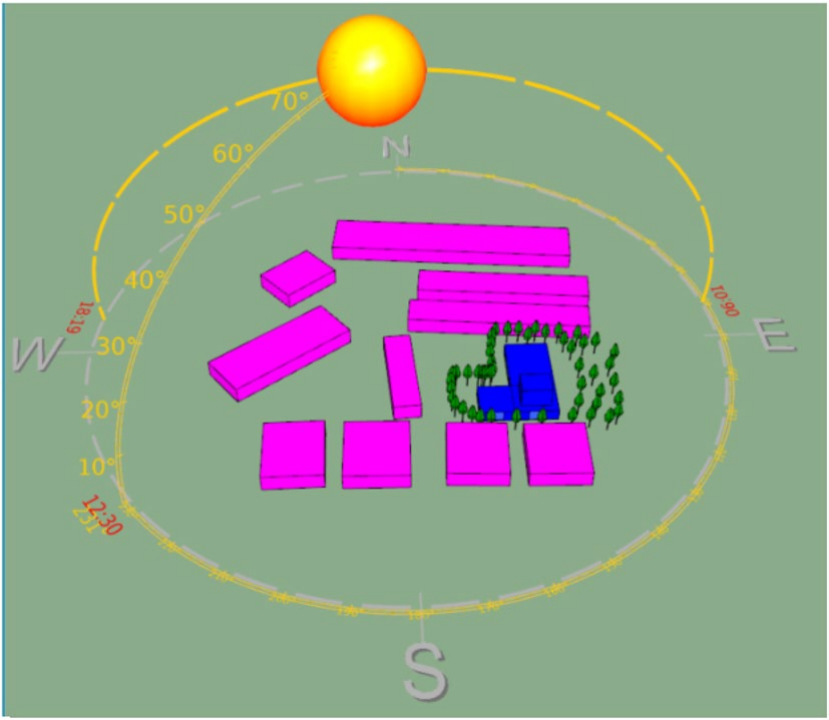
Simulated image of the site. The image was created using SunCast module of IES VE 2023 (Integrated Environmental Solutions Virtual Environment; https://www.iesve.com/software/virtual-environment/applications/solar-shading/suncast).

The experimental setup was established at Anbagam Homeless Shelter in Otteri, West Chennai, Tamil Nadu, India. This location was selected due to its positioning in one of the most densely populated areas in Chennai, with an approximate population density of 30,000 individuals per square kilometer. Conforming to the criteria outlined by the census of India for characterizing an urban area, this locality fulfills the prerequisites of a minimum population of 5000, at least 75 percent of the working population engaged in non-agricultural occupations, and a population density surpassing 400 persons per square unit. The heightened degree of urbanization in this area gives rise to elevated temperatures, commonly associated with the urban heat island (UHI) effect. CRC (Chennai Resilience Centre) collaborated with a homeless shelter, Anbagam, catering to women with psychosocial challenges, to establish a rooftop garden spanning approximately 1000 square feet. The project involved the installation of around 185 mobile vegetable garden kits. Anbagam is situated in Otteri, a neighborhood located in Purasaiwakkam, in the Western part of Chennai. Figure 1 illustrates satellite and simulated images of the site. The selection of this specific location by CRC was driven by the objective of conducting a case study to assess the heat mitigation impact of a rooftop garden.

### Dataset preparation

During the installation of the measuring instruments in April 2023, the terrace garden situated along the eastern wall of the building was already well-established, covering an area of approximately 1000 sq. ft. with 184 grow bags, ensuring sufficient green density. To prevent water seepage from the planter bags and avoid the area behaving as a thermal mass, the planter bag area and the exposed eastern wall stretch were covered with empty white plastic bags made of polypropylene polymer.

### Experimental setup

Data collection involved the installation of two distinct sets of temperature and humidity sensors (AM2306 and AM2105A) designed for industrial use. These sensors comprised enclosed units for outdoor measurements and exposed units for indoor regions. One set of sensors was positioned in the reference monitoring area, which lacked a garden, while the second set was placed in the monitoring area with the garden designed for heat mitigation.

**Figure 2:**
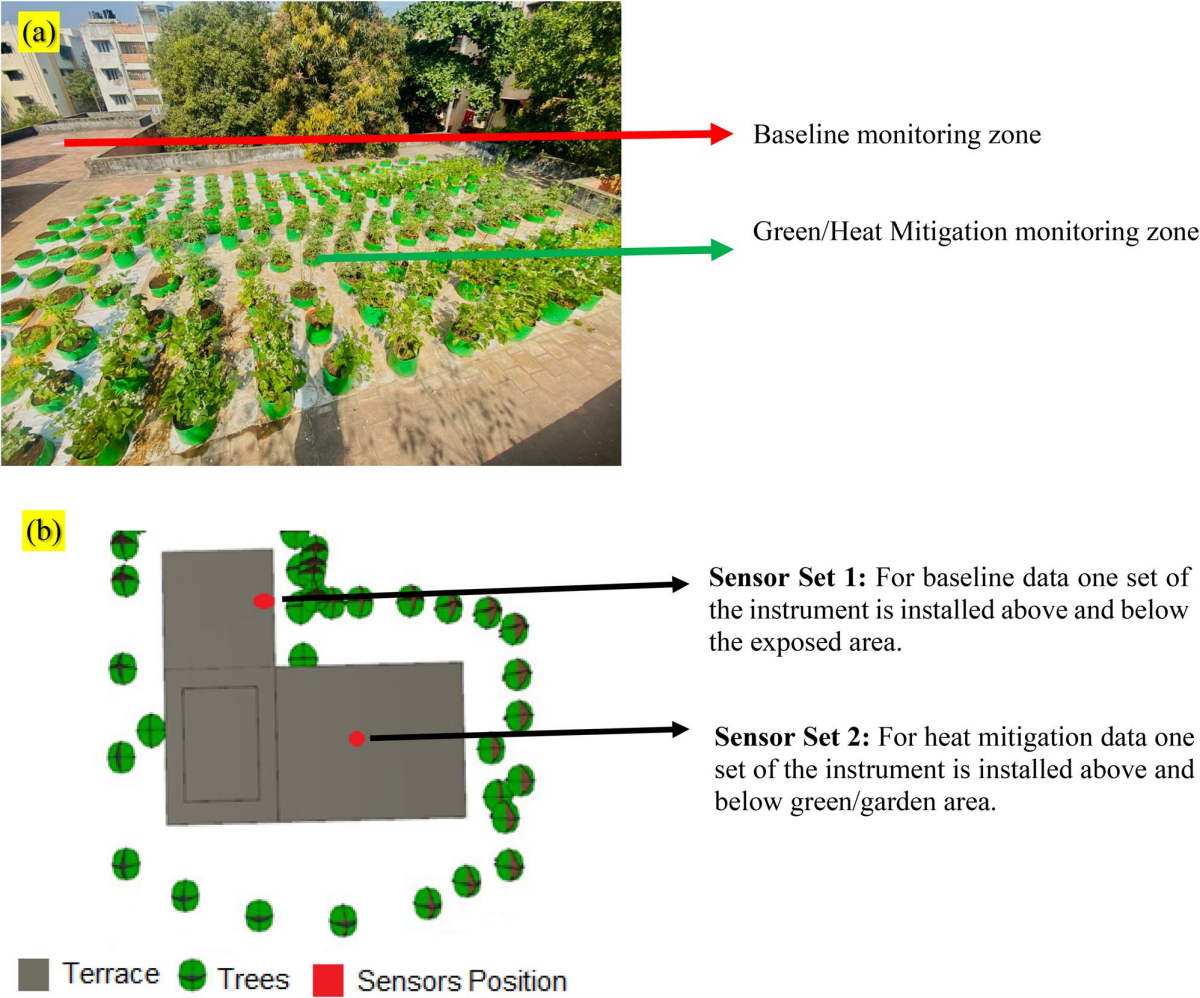
(**a**) Baseline and garden sites used for experiments. (**b**) Sensors locations at garden rooftop. The image (**b**) created using SunCast module of IES VE 2023 (Integrated Environmental Solutions Virtual Environment; https://www.iesve.com/software/virtual-environment/applications/solar-shading/suncast).

**Figure 3:**
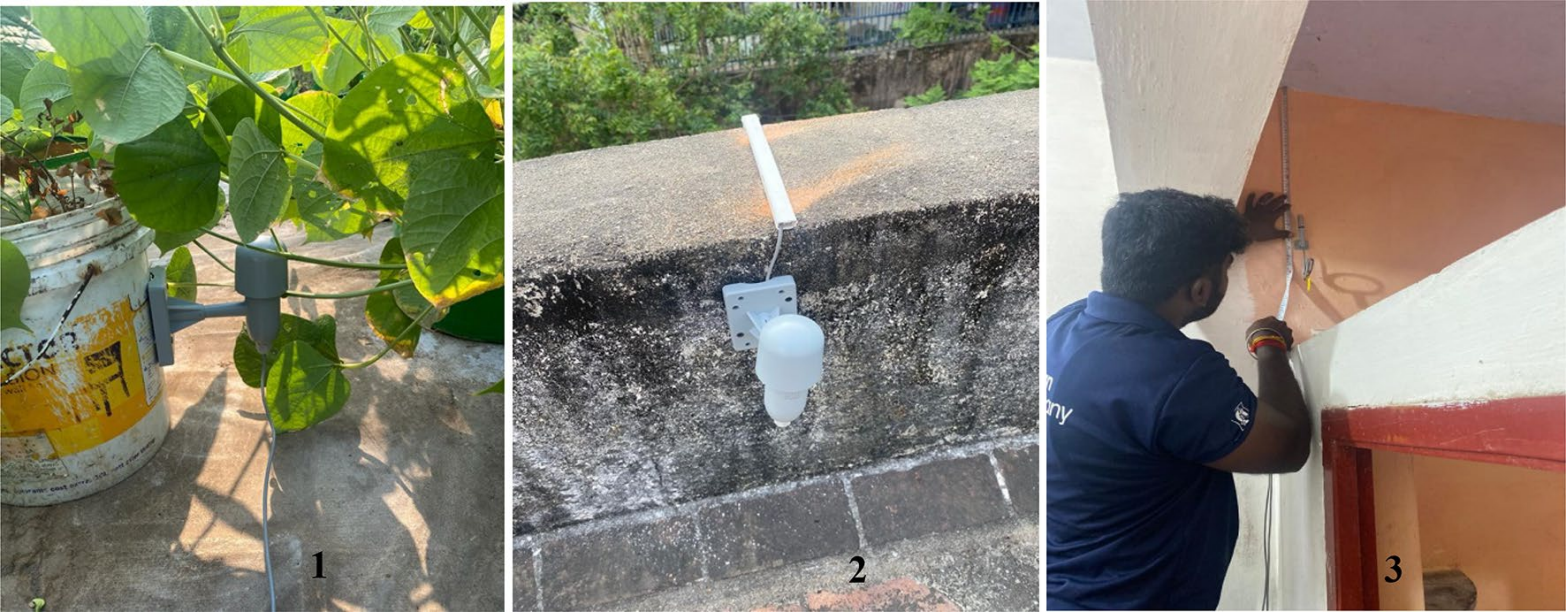
A sensor in the garden area (1), a sensor in the exposed area (2) and a sensor in one of the internal spaces (3).

The municipal administration of Chennai, Tamil Nadu, actively promotes rooftop gardening initiatives and provides logistical assistance. A comprehensive cultivation endeavor was conducted under the aforementioned auspices, encompassing the growth of 184 plants within grow bags filled with a loamy clay soil medium. The soil utilized in the grow bags exhibited a pH level of 6.5, and seeds distributed by the Tamil Nadu Government as part of the Urban Horticulture Development scheme were employed for cultivation. Various seeds, including those of Brinjal, Tomato, Chili, Okra, Cluster Beans, Bush Beans, Radish, Amaranthus, and Coriander, were sown in these grow bags to facilitate cultivation. The use of plant parts in the study complies with international, national, and/or institutional guidelines. Irrigation was administered on a daily basis due to the absence of shading, and growth was facilitated primarily through natural sunlight. Monthly applications of organic vermicompost were employed to provide nutritional support, while a bio pesticide derived from chili was routinely sprayed on the foliage to deter pests. At the time of sensor data collection, the plants ranged in age from 10 to 15 months, exhibiting robust foliage that covered the terrace floor extensively. Hourly sensor data was collected from an area totaling 1000 square feet. A visual representation of this setup can be found in Figs. 2 and 3.

### Programme and calibration

**Figure 4:**
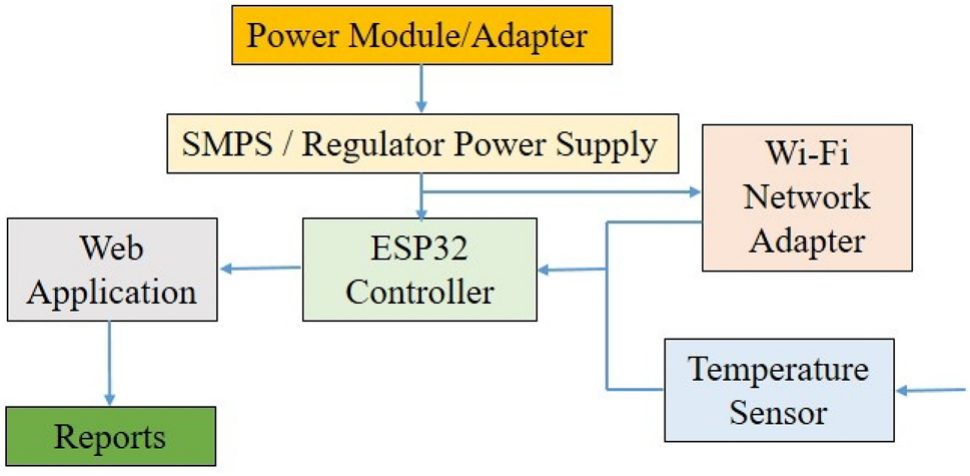
The data recording system used.

**Figure 5:**
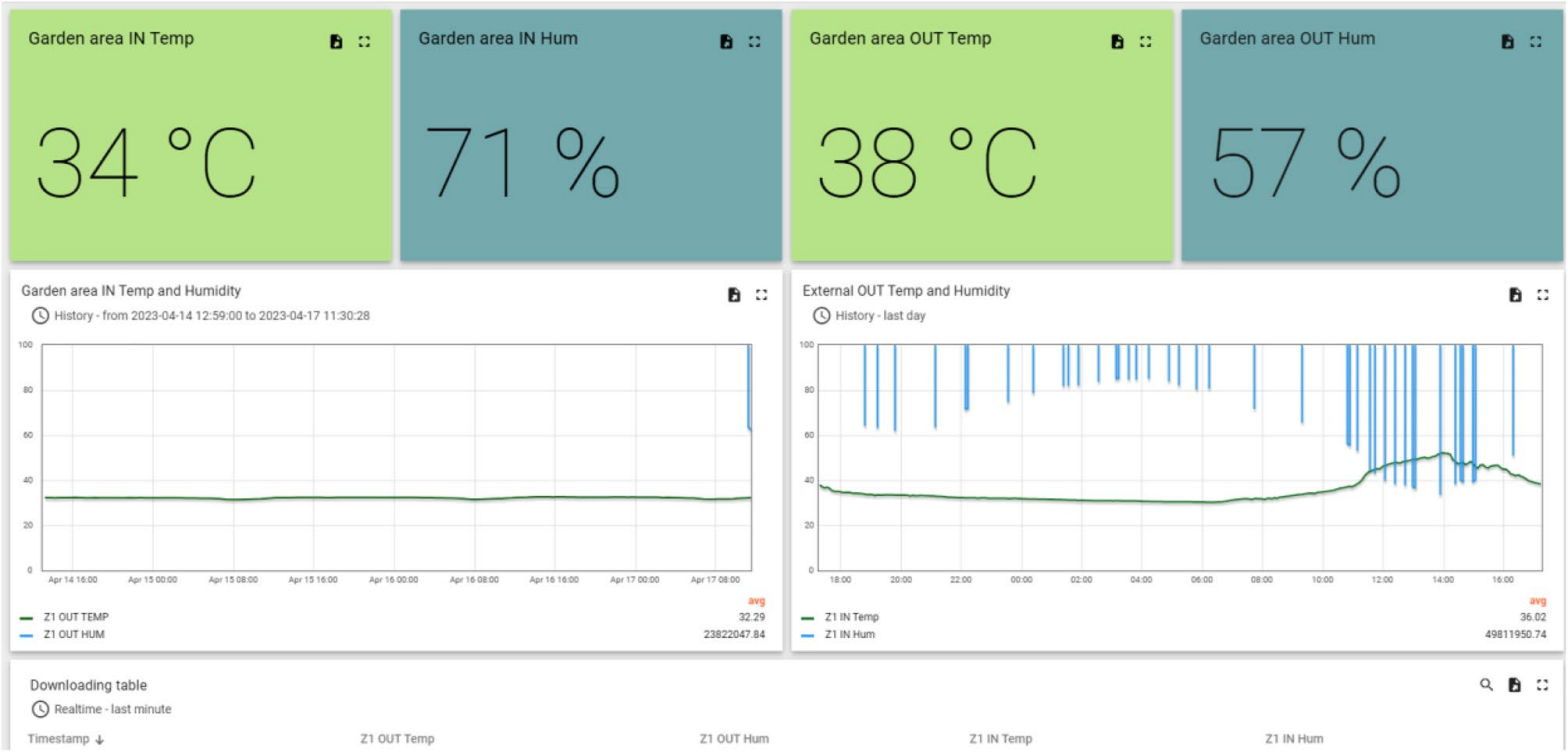
Snapshot of data dashboard.

A straightforward system was employed to process and calibrate the data collected by the sensors. A microcontroller programmed based on the manufacturer’s instructions was used to read the data signals. The controller interpreted the signals and processed the data using MATLAB, a web application. The processed data points were then transmitted via Wi-Fi to a cloud application that had been configured for this purpose. Calibration of the sensors was not necessary unless any discrepancies or mismatches were observed in the collected data. However, a new Wi-Fi dongle was installed to ensure consistent reporting intervals for the data. To ensure data accuracy, the team intermittently used a handheld temperature measurement device to cross-check the recorded data and performed calibration if required (refer to Fig. 4 for a flowchart illustrating the recording system’s architecture). Figure 5 describes the dashboard.

### IES VE model description

The IES VE simulator utilizes data collected from the project site for simulation purposes. Within the Energy modeling, various components are integrated into the model's configuration. ModelIT is employed for generating the 3D model, SunCast assists in understanding sun position-based heat gains and generating thermal images. Additionally, MacroFlo is utilized to consider passive cooling attributed to wind movement, while ApacheSim conducts simulations. Finally, VistaPro is employed for analyzing the results obtained from these simulations.

### Modelling methodology

The facility was modelled to reflect actual openings and window-to-wall ratios. A brick wall with plaster featuring a U value of 2.2 W/m^2^·K was incorporated. For the exposed area, a building with a Solar Reflective Index (SRI) of 16.5 (corresponding to weathered concrete) and no greenery was simulated. Conversely, for the garden area, a building with 190 planter bags with uniform interspatial distance was modelled. These planter bags, filled with soil, were represented as rooms with a soil bed thickness of 300 mm and the foliage via plants was modelled as a roof with both shading impact and an SRI of 50 (corresponding to green)^27^.

A location-specific weather file was generated using the Big Ladder Elements tool, with weather station data sourced from the Visual Crossing tool (station ID: 43,278,099,999). The building model accounted for orientations, window-to-wall ratios, openings and closings at room level, thermal loads (people, light, and ceiling fans), and opening/closing schedules for doors as per actual site operations.

The thermal conductance of the soil medium was adjusted to match the internal temperature reported by sensors for a given day, calibrating the building roof's thermal performance to closely match indoor temperatures. The roof with soil bed had a thermal conductance ranging from 0.4 to 0.7 W/m·K (corresponding to U values ranging between 1 and 1.6 W/m^2^·K).

Upon calibration, peak day temperature matches were achieved for both the garden area and the exposed area. The calibrated values included:

- Concrete: U value of 4.7 W/m^2^·K, SRI 16.5
- Planter bags: U value of 1.48 W/m^2^·K, SRI 50, underlying concrete U value = 4.7 W/m^2^·K
- K value was finalized at 0.62 W/m·K corresponding to a U value of 1.48 W/m^2^·K.

The baseline simulation scenario involved removing all planter bags to establish internal temperatures corresponding to a completely exposed roof. In the proposed case, the planter bags were applied to the exposed area, and simulations were repeated with the U values and SRI values associated with the planter bags and foliage.

Furthermore, simulations were conducted by replacing the planter bags with a uniform soil bed across the terrace with a soil depth of 600 mm (U value ranging from 0.59 to 1 W/m^2^·K), resulting in a temperature difference of 14 °C between baseline and proposed cases.

## Results

In this section, we present the findings obtained from the study conducted by the Chennai Resilience Centre (CRC). The data captured, analyzed, and interpreted for the period from April 1st to June 6th, 2023 is presented. Throughout this duration, the rooftop garden was well-maintained, and all sensors remained functional. While the average difference in room temperatures between the garden room and exposed room across the entire period is reported, specific attention was given to data from particular days identified as the hottest day or a rainy day within this timeframe.

The following dates were considered for further assessment:

- 2nd June 2023: Peak Heat Day—Maximum recorded temperature reached 40 °C at 2:30 PM.
- 2nd June 2023: Peak Real Feel Day—Maximum "real feel" temperature recorded was 55 °C at 3:00 PM.
- 10th May 2023: Rainy Day—Maximum temperature recorded during rainfall was 31.40 °C at 10:00 PM.
- 10th May 2023: Rainy Day—Maximum "real feel" temperature recorded during rainfall was 49.9 °C at 10:30 PM.

In addition to presenting the recorded data, a series of scenarios were generated based on the compiled datasets. We report two specific scenarios as follows:

- **Scenario 1:** Peak heat day—no roof treatment—exposed and weathered concrete surface.
- **Scenario 2:** Peak heat day—whole roof layered with white cement bags followed by planter bags with foliage.

## Summary of recordings and results

Based on the data collected from April 1st to June 6th, 2023, several general observations have emerged:

- During Sunshine hours:
  The temperature in the exposed areas, on average, is 2–3 °C higher than the reported temperature for the locality.
  The temperature in the garden region is 1.5 °C lower than the reported temperature for the locality.
  The room under the plantation experiences temperatures 2–3 °C cooler, on average, than the exposed room.
- Post sunset till sunrise:
  The temperature in the exposed areas is on par with the reported temperature for the locality.
  The temperature in the garden region is 1.5 °C higher or on par with the reported temperature for the locality.
  The temperature of the room under the garden is, on average, comparable to the exposed room.

These findings provide valuable insights into the thermal dynamics and performance of the rooftop garden in mitigating heat and improving the indoor environment within the shelter.

## Case studies

### Case Study 1: Current scenario – roof covered partially with white cement bags and planter bags with foliage in the Northeast section on the hottest day

**Figure 6:**
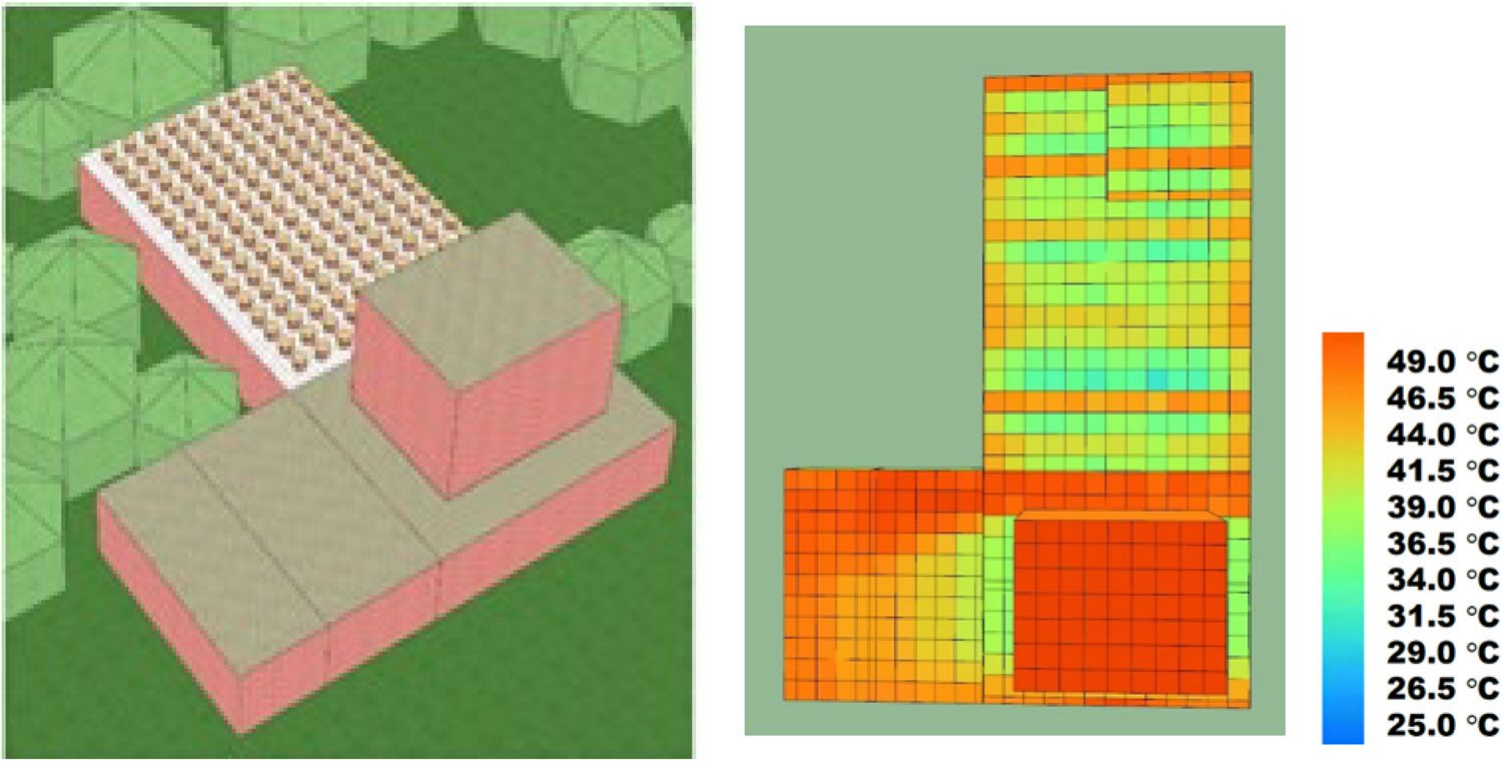
Current configuration at Anbagam shelter home and heat map visualization. The heat map was created using SunCast module of IES VE 2023 (Integrated Environmental Solutions Virtual Environment; https://www.iesve.com/software/virtual-environment/applications/solar-shading/suncast).

As depicted in Fig. 6, thermal imagery analysis reveals that the average surface temperature in the external garden area reached 40 °C, while the average surface temperature of the exposed roof space was measured at 45 °C. This comparison highlights the significant difference in temperature between the garden area and the exposed roof space.

**Figure 7:**
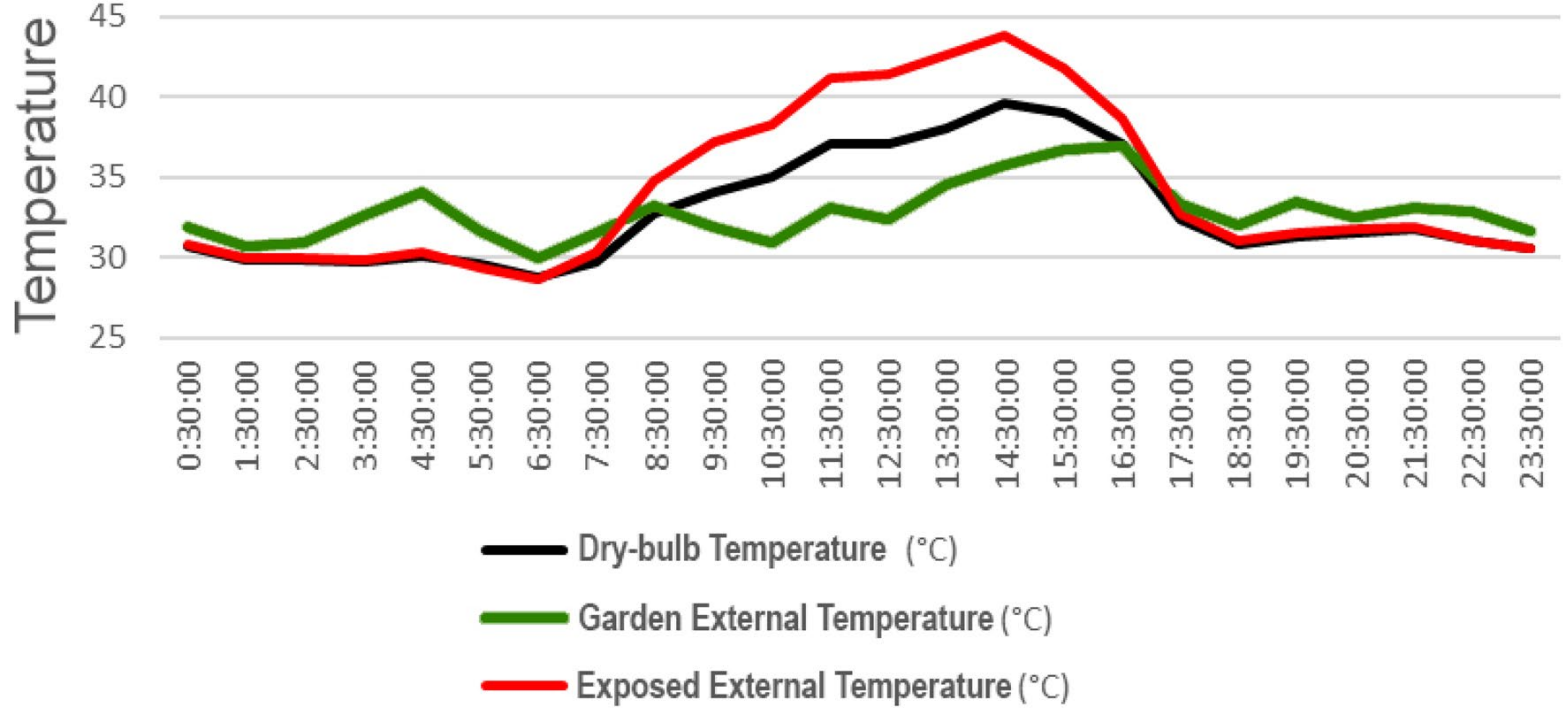
Comparison of observed external temperature in Otteri area with exposed surface and garden surface temperature over time (hourly).

**Figure 8:**
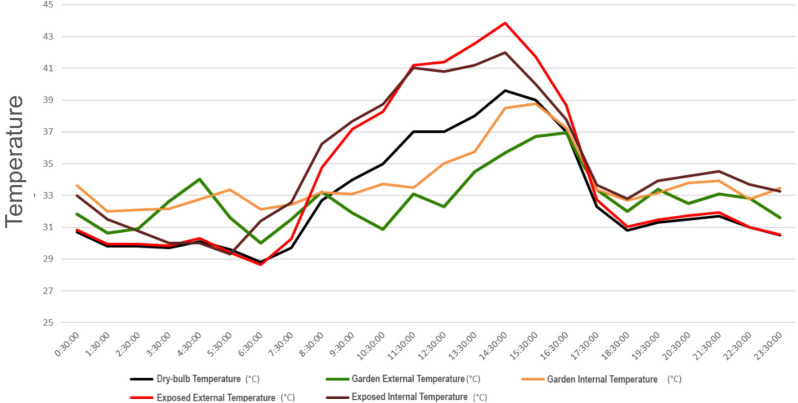
Comparative analysis of temperature within the building, terrace, and neighborhood.

**Figure 9:**
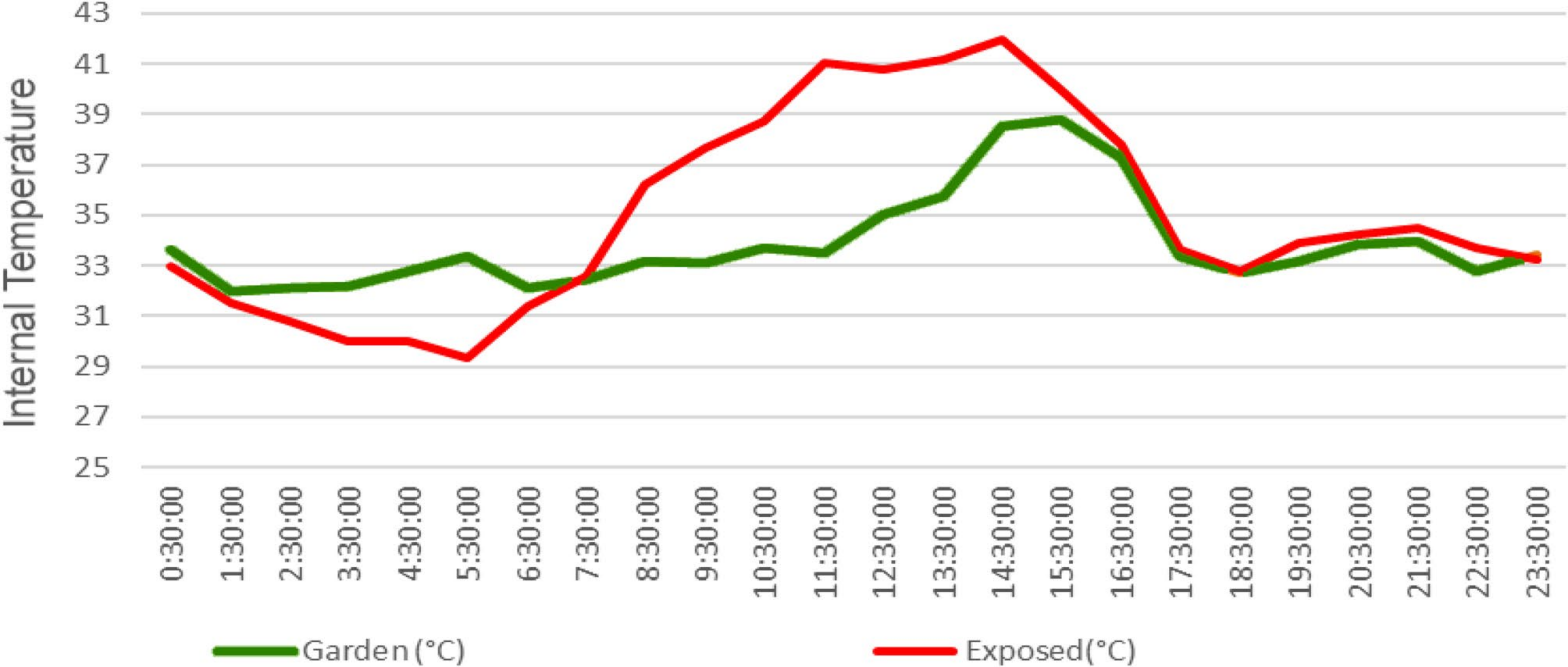
Temporal comparison of temperatures in rooms below the garden and exposed terrace.

1. Assessment of weather data and external surface temperature:

In Fig. 7, the temperature difference between recorded weather data and the exposed and garden-covered roof space is presented. The following observations can be made:

- During sunshine hours (6 am to 6 pm), the temperature in the garden or plantation area is lower than the externally recorded temperature for the Otteri neighborhood (referred to as dry-bulb temperature in subsequent graphs) and the exposed roof space. This is attributed to the shading effect of the plants through foliage and the presence of the white cement bag under the planter bags, which mitigates heat absorption.
- Post sunset (6 pm) till 8 am, the temperature in the garden area is higher than the recorded temperature for Otteri and the exposed roof temperature. This can be attributed to the release of trapped heat from inside the building, which moves upward through the ceiling to the garden area. The exposed terrace surface, on the other hand, cools faster as there are no planter bags obstructing contact with the atmosphere. Additionally, there is a heat build-up in the store room below the garden due to lack of ventilation and closed doors, compared to the room below the exposed roof surface, which benefits from open windows allowing for better ventilation and heat dissipation.

2. Assessment of Surface Temperature and Internal Temperature:
  The findings presented in Fig. 8 illustrate the temperature variations between the Otteri Neighborhood, external roof sites, and the corresponding rooms below. Figure 6 highlights distinct patterns in internal temperature within the building for the rooms under investigation.
  In the exposed room (or the room below the exposed terrace space), the temperature is consistently lower than the corresponding temperature of the exposed roof surface. This can be attributed to the ventilation in the internal space, allowing heat to dissipate to cooler surrounding rooms.
  In the garden room (or the room below the garden), the temperature is generally higher than the external garden surface temperature. This is due to heat transfer through internal partitions, as this room is located directly beneath the rooftop garden. These findings underscore the intricate relationship between temperature, roof cover, ventilation, humidity, and the structural dynamics that collectively influence the heat experience.

3. Assessment of Roof Temperature in Exposed and Garden-Covered Portions:
  The temperature difference between the rooms below the garden and exposed terrace space is illustrated in Fig. 8. Observations from Fig. 9 indicate the following:
  During the period from midnight to 7:30am, the temperature in the room below the garden is higher than the temperature in the room below the exposed terrace space. This can be attributed to the closed doors and lack of windows in the storeroom, which restrict ventilation and allow for the accumulation of heat.
  However, when the storeroom doors are opened at 7:30am, a reverse trend is observed. The temperature in the storeroom becomes significantly lower than the temperature in the room below the exposed surface, particularly during the hottest hours of the day. This could be due to the insulative effect of the roof garden in conjunction with the open doors allowing for heat evacuation during this period.

### Case-study 2: Current scenario – roof covered partially with white cement bags and planter bags with foliage in the Northeast section on a rainy day

**Figure 10:**
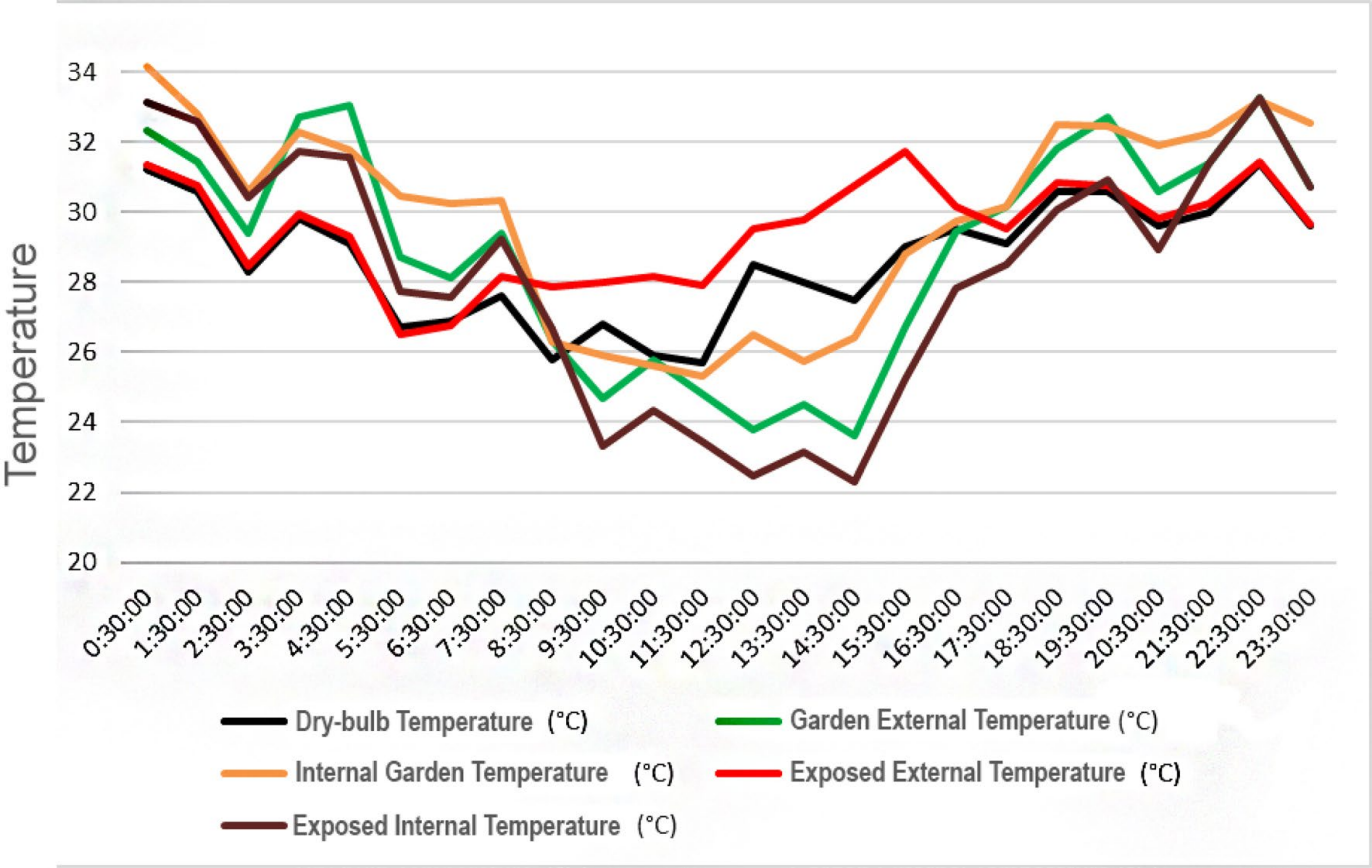
Temperatures on a rainy day: Otteri neighborhood, roof, and rooms below.

An analysis of external and internal temperatures is presented based on the data recorded on May 10th, 2023, which was identified as the wettest day within the study period. The assessment of temperature in the room below the garden and the exposed roof surface is presented in Fig. 10, showcasing the temperatures recorded on a rainy day during the study period in the Otteri neighborhood, on the roof, and in the rooms below. The x-axis represents time, while the y-axis represents temperature. From Fig. 10, the following observations can be made:

- Intermittent rainfall occurred on May 10th, from 5:30 AM to 3:30 PM, coinciding with a general decrease in temperature in the Otteri neighborhood (referred to as dry-bulb temperature). During these rain episodes, heat trapped inside the building was released, leading to spikes in the temperature of the exposed roof surface when the rain subsided.
- The garden area maintained a cooler temperature throughout this period, and variations in temperature were monitored.

### Case-study 3: Average temperature difference between the rooms during the entire study period

**Figure 11:**
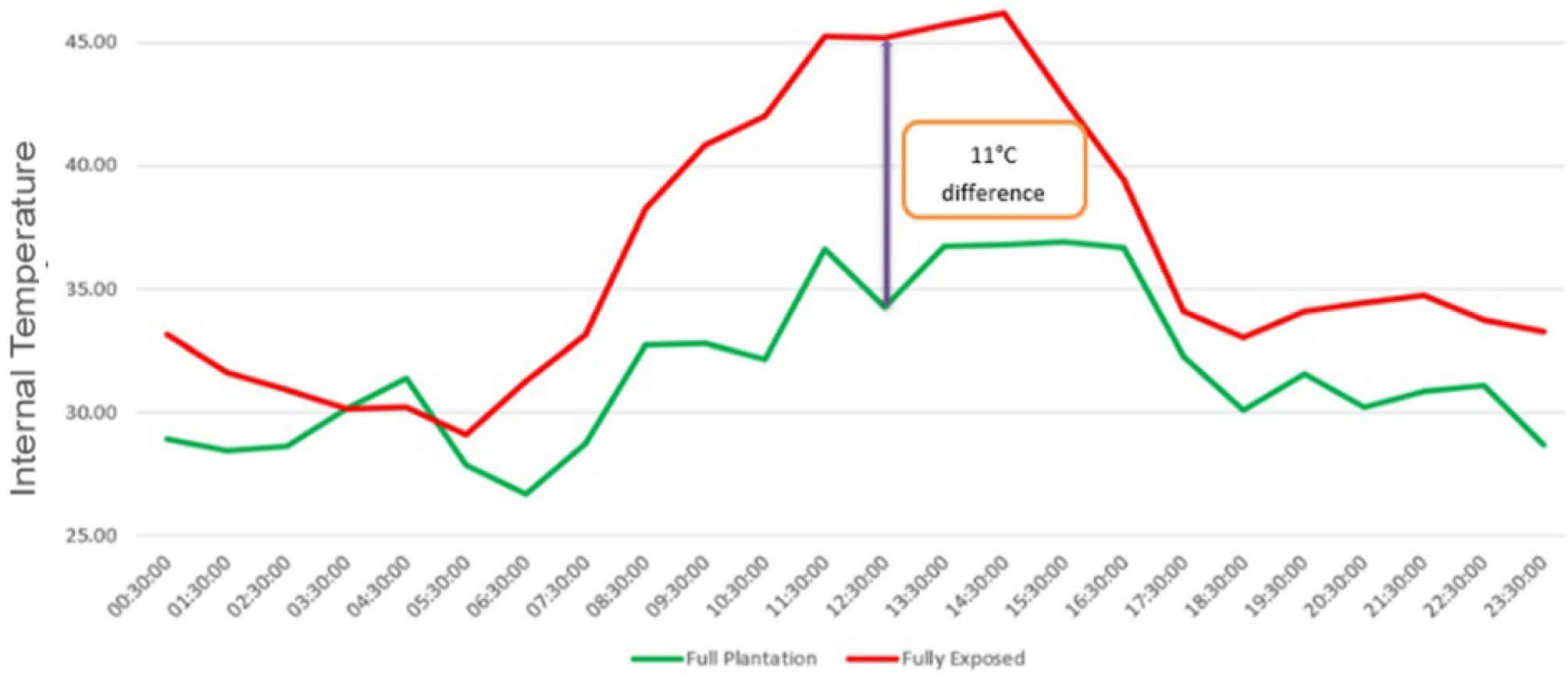
Average temperature difference between room under the garden and exposed roof space over time (°C).

Figure 11 illustrates the average temperature difference between the room below the garden and the exposed roof space throughout the entire study period, spanning from April 1st, 2023, to June 6th, 2023. The figure highlights that the peak average temperature difference between the two rooms is 5°C, occurring around 12 noon. This means that during the hottest time of the day, the temperature in the room below the garden area is consistently up to 5°C lower than the temperature in the room below the exposed roof space.

### Case-study 4: Simulated scenarios that compare a fully exposed roof space and a roof completely covered with planter bags

The data presented in this study is based on actual readings derived from the project site. These simulations performed on IES VE® aim to provide an estimated representation of the potential impact of a fully covered roof with grow bags compared to a roof with no grow bags. The analysis focuses on the hottest day of the study period, June 2nd, 2023, for the two scenarios. The IES VE model was developed, and the roof U values and reflective indices were adjusted to correspond with the temperature data collected from the sensors. These adjusted models were then used to simulate scenarios with 100% exposure and 100% plantation conditions, aiming to determine the temperature differences between the baseline and proposed conditions. The process of model creation included aligning the roof U values and reflective indices with the temperature readings from the sensors. Subsequently, this adjusted model was utilized to conduct simulations under both 100% exposed and 100% plantation conditions, estimating the temperature variations between the baseline and proposed scenarios.

**Figure 12:**
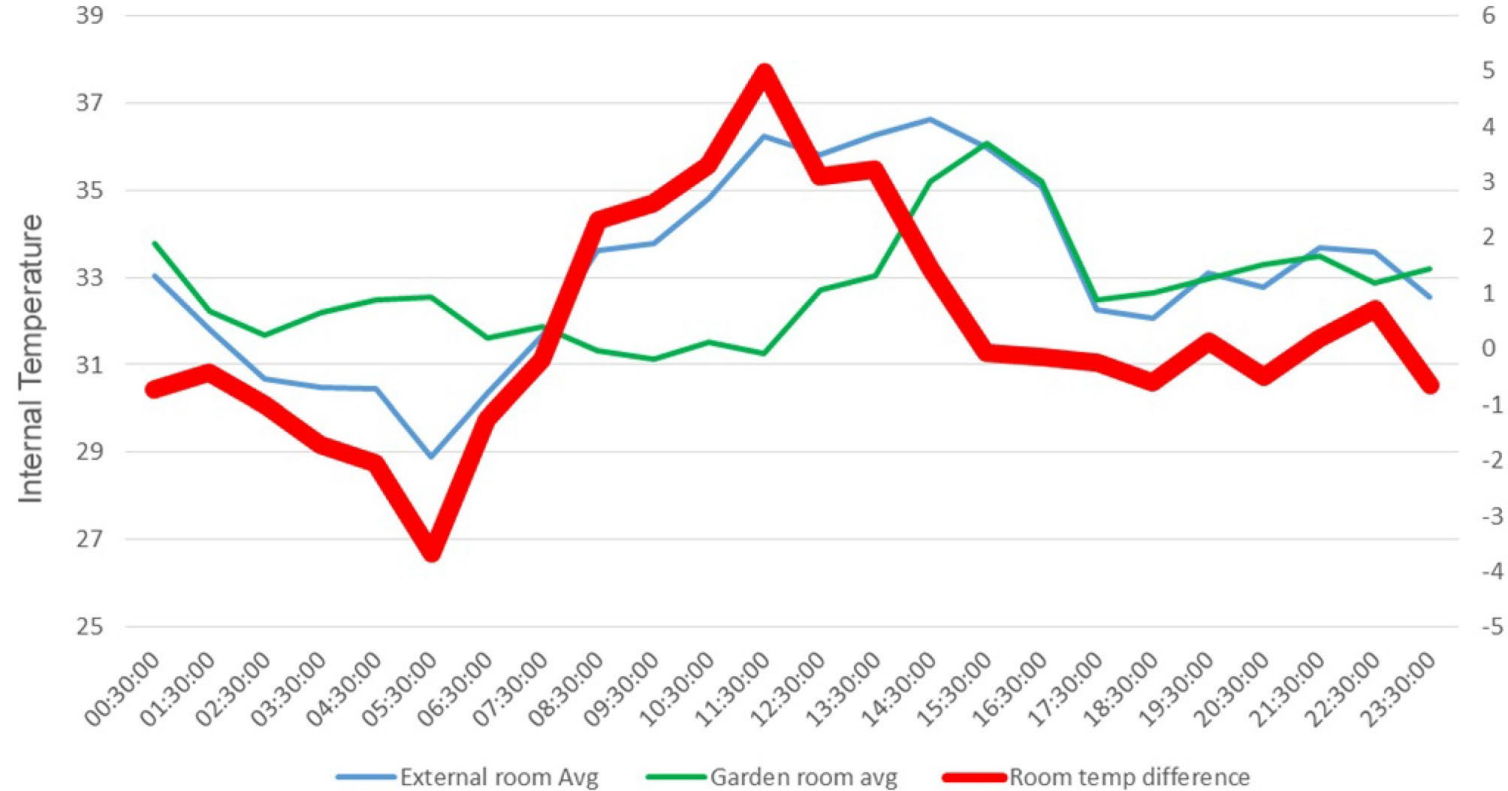
Comparison of external temperatures: roof fully covered by garden vs. roof fully exposed and without garden.

Figure 12 provides a visual illustration of the likely internal temperature difference between the two roof spaces.

Key findings from the simulations include:

- The internal temperature within the building is consistently lower when the roof is fully covered with a garden compared to when the roof is completely exposed during summers.
- The hottest day temperature difference between the two scenarios is approximately 11 °C, indicating a significant relief of around 4 °C lower temperature when compared to the current setup where only a portion of the roof is covered with plantation.
- It is important to note that the peak temperature difference in the simulation occurs around 11:30am due to heat exchange between the rooms. However, in a hypothetical scenario with two independent buildings (one with a roof garden and one without), where there is no internal heat transfer, the peak temperature difference would be observed at 3 pm and could reach up to 14 °C. This discrepancy is attributed to the learning data set from the sensors, which indicates the peak differential occurring around 11:30am to 12 noon. In real-time scenarios, the peak temperature difference typically occurs around 2:30 pm to 3 pm.

**Figure 13:**
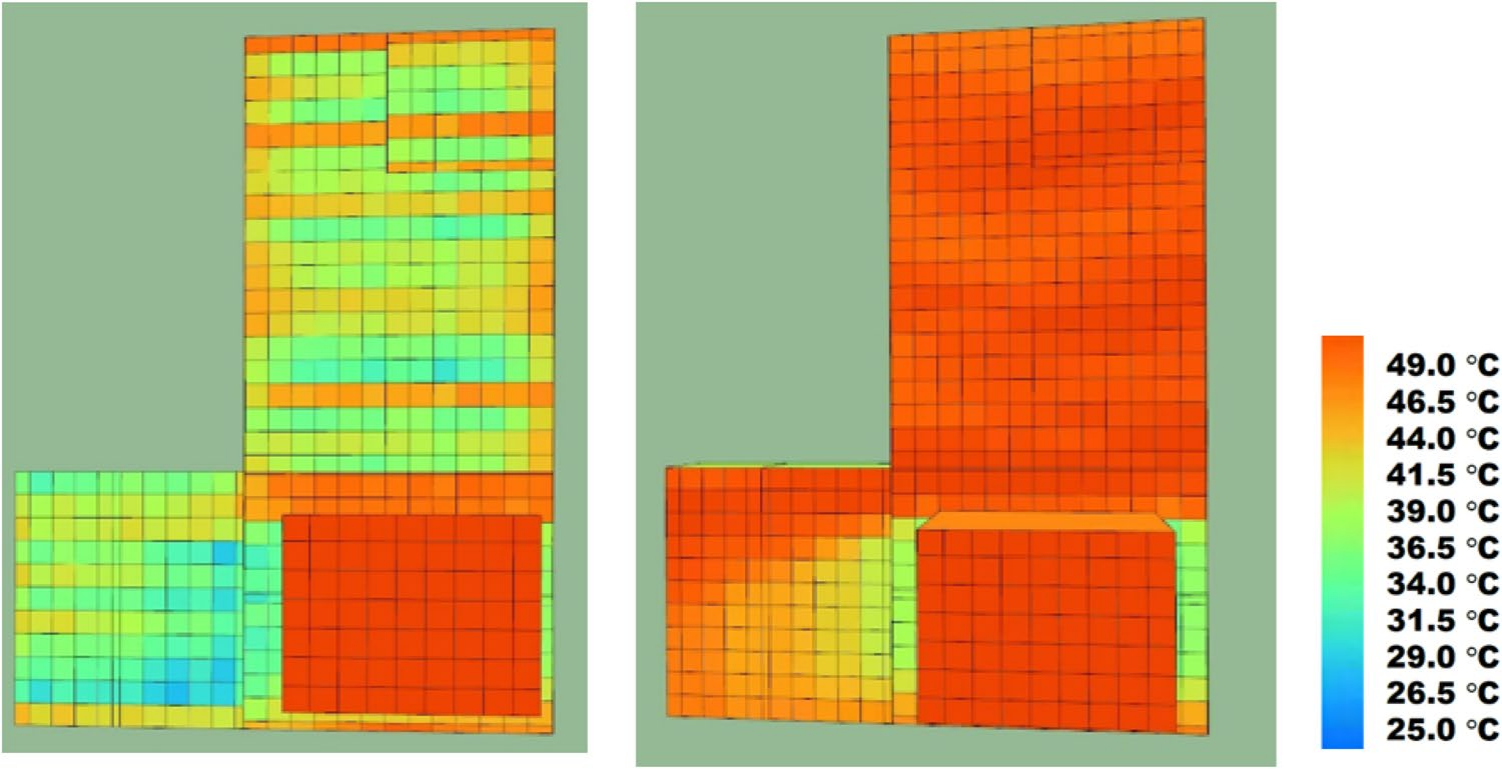
Thermal images of the spaces: fully covered roof (left) and fully exposed roof (right). The heat map was created using SunCast module IES VE 2023 (Integrated Environmental Solutions Virtual Environment; https://www.iesve.com/software/virtual-environment/applications/solar-shading/suncast).

Figure 13 presents a visual representation of the simulated scenarios. The images depict that the peak surface temperature on the roof is approximately 38°C when the entire terrace is covered with grow bags, whereas it reaches around 49°C when the entire roof is exposed.

## Observations

From the Figs. 7, 8, 9, 10, 11, 12 and 13, a notable distinction emerges: the most effective outcomes are observed when the UHI mitigation is implemented across the entire roof, challenging the assumption that applying the solution to isolated sections would yield comparable results. Two key observations from the study support this:

A. The temperature difference is maximized at 7.5 degrees Celsius when the solution is applied to a specific section at 11:30 AM.
B. Conversely, if the same mitigation strategy is applied uniformly across the entire roof, the estimated temperature difference increases to at least 11 degrees Celsius, particularly around 1 PM.

The simulation models, derived from calibrated data, reveal a critical insight—the heat absorbed by the exposed roof is transmitted to the room with the mitigation strategy applied. This room acts as a cooler zone, illustrating the basic principle of heat flow from hotter to cooler zones.

Consequently, our study underscores that the effectiveness of UHI mitigation is contingent on the comprehensive application of strategies, requiring active participation from all stakeholders. Lowering building roof temperatures not only enhances thermal comfort but also contributes to substantial energy savings. When scaled up, the cumulative impact of such strategies across multiple buildings can significantly reduce local temperatures. Extrapolating further, the concerted efforts of multiple localities can contribute to restoring city-level temperatures to levels reminiscent of many years ago.

In essence, our findings challenge the conventional approach of applying UHI mitigation strategies to specific sections, advocating for a holistic, city-wide approach for optimal and sustainable results.

Based on the case studies, the following observations are made:

1. Covering the entire roof with planter bags and promoting foliage leads to a reduction of 4 °C to 11 °C temperature in indoor thermal comfort beneath the garden rooftop as compared to a completely exposed roof setup.
2. Through simulation, it is observed that this temperature difference can potentially reach up to 14 °C (under ideal conditions) if the planter bags are replaced with a soil bed of 2–3 feet depth and dense foliage of plants. Therefore, it is recommended to cover the entire terrace space with a garden on a soil bed for optimal results. However, it may not always be practically feasible. In such cases, using grow bags, as in the current setup, to cover the entire terrace space can still yield favorable outcomes.

## Limitations

This section discusses the limitations of the proposed work.

1. *Air circulation and cross ventilation*: The regulation of temperature within the facilities is influenced by factors such as air circulation, cross ventilation, and heat gain through facades. While our study measured temperatures at the terrace and room levels, a more accurate analysis could have been achieved by capturing data on air changes and wall heat gain. This additional data would have facilitated a more precise isolation of heat gains from the roof.
2. *Uniform assumption for air circulation*: Given the primary focus of estimating the impact of a roof garden in mitigating the urban heat island (UHI) effect and understanding the effectiveness of the mitigation strategy on a section of a larger roof, we made the assumption that air circulation is common and uniform throughout the facility. This assumption is based on the interconnected nature of rooms with multiple openings. Additionally, the presence of trees covering facades led us to consider heat gain through walls as uniform.
3. *Height limitation for sensor placement*: The facility, being a home for the mentally challenged, posed a unique challenge in sensor placement. To ensure safety and accessibility, sensors had to be positioned at heights exceeding 6 feet, as most of the inmates cannot reach them. This limitation impacted the number of deployable sensors and, consequently, the granularity of data collection.

Despite these limitations, our study aimed to fulfill its core objective of assessing the impact of a roof garden in mitigating the UHI effect within the defined constraints.

## Conclusion and future work

Our case study highlights the significant impact of terrace gardens utilizing grow bags as an effective strategy for mitigating urban heat island (UHI) effects and enhancing building climate resilience. Through the implementation of planter bags on rooftops and the cultivation of lush foliage environments, our findings demonstrate a substantial reduction in internal temperatures, ranging from 4 to 11 °C, compared to fully exposed roof setups. This enhancement in thermal comfort not only addresses immediate heat-related challenges but also bolsters the overall resilience of buildings in response to escalating temperatures. Our simulation results further indicate that the potential benefits of UHI mitigation can be amplified by adopting an evenly distributed soil bed with dense foliage, potentially leading to an even greater temperature reduction of up to 14 °C. However, practical considerations and logistical constraints may favor the continued utilization of grow bags as a means of covering entire terrace spaces.

For future research endeavors, we advocate for conducting more extensive and rigorous studies on terrace gardens with grow bags. This entails exploring different localities and building structures to capture the diverse range of impacts that green infrastructure can have on UHI mitigation and building climate resilience. By expanding the scope of research, a more comprehensive understanding of the benefits and intricacies of terrace gardens can be attained, thereby contributing to the development of effective strategies for creating sustainable and resilient urban environments.

## Data Availability

The datasets generated and/or analysed during the current study are not publicly available due to experiment protocol approved by the Institutional Board, but are available from the corresponding author on reasonable request.
